# Palliative Care in the state of Rio de Janeiro (Brazil): characteristics of the services

**DOI:** 10.25122/jml-2023-0083

**Published:** 2023-08

**Authors:** Carolina Neiva Guedes da Silva, Fernanda de Sousa Pousa, Tulio Loyola Correa, Ricardo Tavares de Carvalho

**Affiliations:** 1 Division of Palliative Care, Instituto Paliar, São Paulo, Brazil; 2 Hospital das Clínicas, Faculty of Medicine, Universidade de São Paulo, Sao Paulo, Brazil

**Keywords:** palliative care, end-of-life care, medical assistance, health services, Brazil, Latin America

## Abstract

This research explores the status of Palliative Care (PC) services in the state of Rio de Janeiro (RJ), Brazil, by identifying the number of services and their respective characteristics. Through an observational, cross-sectional, descriptive, and quantitative approach, this study examines a textual and numerical database about professionals who provide palliative care assistance in Rio de Janeiro. Out of the existing palliative care services analyzed, fourteen were evaluated, twelve of which were located in the RJ metropolitan region. The majority of professionals worked with private funding within hospitals. The PC teams were most commonly composed of physicians, with only 17.1% of the professionals having formal PC training. Palliative Care services in Rio de Janeiro seem to follow a similar pattern to Brazil and Latin America. However, the characteristics of this pattern may not adequately address the significant needs of middle-income countries.

## INTRODUCTION

In 2014, the World Health Assembly approved resolution 67.19, which urged countries worldwide to integrate Palliative Care (PC) as an essential aspect of universal health coverage [1]. However, six years later, the inequality in accessing PC remained evident, particularly in low- and middle-income countries, which account for 76% of the PC demand [2]. This inequality is also evident in Latin America, where there are 1,562 PC teams (2.6 teams per million inhabitants), 59% of them concentrated in just three countries: Argentina, Chile, and Brazil [3].

Brazil is a country of 8,514,877km^2^, divided into 27 federative units, with about 210 million inhabitants and 198 PC teams working mainly in inpatient and outpatient settings [3]. It is classified at the level 3b of PC development by the Worldwide Hospice Palliative Care Alliance (WHPCA) [4]. This signifies a widespread provision of PC services with public and private financial assistance, along with the availability of professional training initiatives by national PC strategies. However, there is still a lack of effective organization in the implementation of public health policies that address these issues [5].

The Brazilian state of Rio de Janeiro (RJ), one of the nation's federative units, covers a total area of 43,696 Km^2^, a population of 17,463,340 inhabitants, and has the second-largest gross domestic product per capita in the country (R$ 44,222.26, equivalent to US$ 8,359,600) [6]. The state records approximately 144,000 deaths per year, with the primary three causes of death being cardiovascular diseases, neoplasms, and respiratory diseases [7]. This epidemiological profile closely resembles that of the world population [8].

In this context, about 2/3 of these deaths should receive PC in the last year of life [9]. In a publication by the National Academy of Palliative Care (ANCP) in 2019, 13 PC teams were identified in the state of RJ [10]. However, only general data (e.g., number of beds, access to opioids, and profile of care) are available [10]. Therefore, this study aimed to provide a more detailed view on the PC services in the state of RJ in 2022.

## METHODS

This research uses an observational, cross-sectional, descriptive, quantitative design following the Strengthening the Reporting of Observational Studies in Epidemiology (STROBE) guidelines to describe the PC services in Rio de Janeiro [11]. The participants were professional coordinators of PC services in the state, which were reached out to during the national PC conference in RJ. A questionnaire was distributed, along with the printed Informed Consent Form (ICF), in a dedicated conference session. Additionally, the questionnaire was sent electronically to those who could not attend the session.

The individuals who refused to complete the questionnaires were excluded from the research. Only the questionnaires filled out by the service coordinator or a substitute indicated by them were considered valid.

The questionnaire (Supplementary Material 1) was developed specifically for this study and based on the most pressing questions and needs at the time. Through multiple-choice and open-ended questions, the instrument covers five significant domains: participant’s data, the service they represent, certain service characteristics, teaching activities, training of professionals in PC, and other relevant information.

The variables analyzed were the location of the service in the state of RJ, nature of services (public or private), place of functioning (inside or outside the hospital), age group served, type of care in PC, number of professionals, workload dedicated to the service, opioid access, teaching activities, and the PC training level of professionals.

After being anonymized, data were analyzed using *Excel 2016* and the descriptive analyses were performed using *SPSS software*, version 26. Unreported data were excluded from the analysis. The results are presented in terms of descriptive statistics, reporting means or percentages.

## RESULT

**Figure 1:**
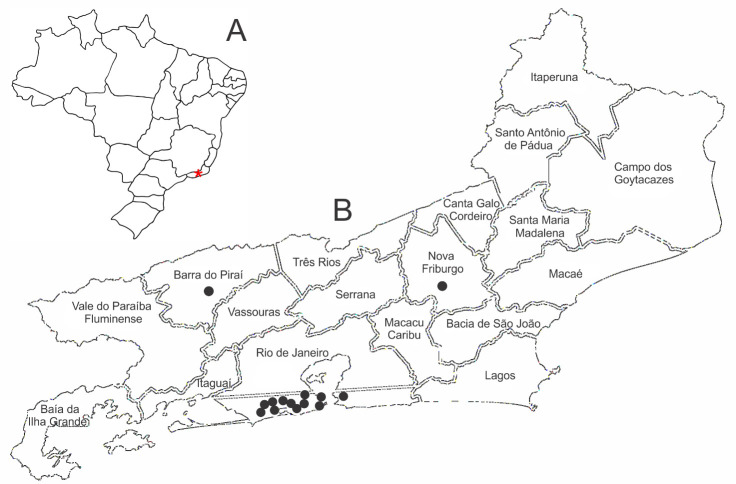
A) Geographical location of RJ state in Brazil; B) Distribution of PC service in the state

All the 14 service coordinators answered the questionnaire, yet in four of the forms, the answers regarding opioid access, teaching activities, and professional PC training were incomplete. Eleven services were located in the same city (Figure 1). The majority of these services were private (57.1%). All services provided hospital care, primarily in highly complex hospitals (71.4%) and without specific beds destinated to PC (78.6%) (Table 1). Only two services offered home care. More than half of the sample worked as a hospital PC consulting group (57.1%). Only five services provided pediatric care in PC, and none of them exclusively provide care to children.

**Table 1:** Characteristics of the Palliative Care (PC) services in the state of Rio de Janeiro (RJ), Brazil (n=14)

Characteristics	n (%)
Located in the capital (RJ)	11 (78.6)
University-based*	5 (50)
**Funding**	
Public	6 (42.9)
Private	5 (35.7)
Insurance	6 (42.9)
**Service modality**	
Hospital	14 (100)
Home care	2 (14.3)
**Modality of care**	
Consultation team	8 (57.1)
Hospital admission	6 (42.9)
Outpatient clinic	7 (50)
Domiciliary	2 (14.3)
**Age group served**	
Elderly	14 (100)
Adults	13 (92.9)
Children	5 (35.7)
Includes non-cancer patients (yes)	11 (78.6)
Specific beds destinated to PC (yes)	3 (21.4)
Presence of volunteers (yes)*	4 (40)
Offers activities aimed at caring for family members/caregivers (yes)*	5 (50)
Offers activities aimed at caring for the PC team (yes)*	6(60)

* n=10

In terms of other specific aspects of care, 50% of the services offered activities dedicated to caring for family members and caregivers of patients, 60% offered activities focused on caring for the PC team, and 40% included volunteers in the service.

Over 80% of the sample experienced no difficulties in accessing opioids, with the most easily available ones being codeine, tramadol, and morphine, while access to methadone was reported by only 60% of the services (Table 2).

**Table 2:** Characteristics of the Palliative Care (PC) services regarding opioid access (n=10)

Characteristics	n (%)
Difficult access to opioids (yes) 2 (20)	
**Available opioids**	
Codeine	9 (90)
Morphine	9 (90)
Tramadol	8 (80)
Methadone	6 (60)
Oxycodone	2 (20)
Fentanyl	5 (50)

Every PC service evaluated included physicians in their personnel. In three of the services, physicians were the only type of professionals (Table 3). In 78.6% of the services there were no nurses with exclusive dedication to PC.

**Table 3:** The number of health care workers by profession in each service

Service	Doctors	Nurses	Nursing assistants	Psych.	Social workers	Chaplain	Nutri.	Phono.	Physio.
**A**	2	0	0	0	0	0	0	0	0
**B**	4	0	2	0	1	0	2	0	2
**C**	3	5	30	2	0	0	5	5	10
**D**	2	3	4	1	1	4	2	1	3
**E**	8	7	23	1	1	1	1	1	4
**F**	2	1	100	1	1	1	10	4	25
**G**	3	0	0	0	1	0	0	0	1
**H**	2	2	0	1	2	0	1	0	0
**I**	1	0	0	0	0	0	0	0	0
**J**	27	56	97	6	9	16	9	0	5
**K**	4	0	0	0	0	0	0	0	2
**L**	4	2	0	2	2	0	0	0	5
**M**	‡	‡	‡	‡	‡	‡	‡	‡	‡
**N**	4	0	0	0	0	0	0	0	0

‡ : not informed; Psych.: psychologists; Nutri.: nutritionists; Phono.: phonoaudiologists; Physio.: physiotherapists

Only 10 services provided data on the PC training of professionals (n=550). Of these, 94 (17.1%) professionals had formal training in PC (postgraduate training with at least 180 on-site course hours), 71 (75.5%) being affiliated to the same service. Half of the services were university-based, but only one provided a PC residency/fellowship training program.

## DISCUSSION

This study showed that the PC services were mostly concentrated in only one (Rio de Janeiro, RJ) of the 92 cities that make up the state of RJ. The distribution pattern of services in our sample (almost 80% concentrated in the capital) is also observed in countries with the same PC development level as Brazil (WH**-**PCA level 3b) [2]. In Colombia, PC services in the department of Antioquia are concentrated in the city of Mendellín (92%) [12], the second largest city in the country in terms of economic importance [13]. In El Salvador, 21 of the 25 PC services are in-hospital consultation services [3]. A similar pattern was observed in the services in the state of RJ, where 71% are exclusively in-hospital consultations.

This trend is also reflected in Brazil, where more than half of the PC services (54.5%) have this characteristic, as well as 44.8% in Latin America [3], suggesting a consistent pattern in the region. Therefore, it is necessary to re-evaluate the strengths and weaknesses of structuring PC care within hospital settings. Meanwhile, developed countries have already emphasized the necessity of including PC in outpatient and primary health care [14].

In addition, our research revealed that more than 70% of the initiatives in RJ did not have the minimum requisites to be considered a PC service according to the World Health Organization (WHO). These requirements include having a full- or part-time physician and a full-time nurse with PC specialization training [15].

In our study, a lack of PC training was observed among the health professionals, with only 28% of them providing full-time assistance for PC services. However, the absence of specific training in PC could be linked to inconsistencies related to the self-characterization of different PC initiatives as a PC service.

Although this research obtained similar results to a national study from 2018 [10], it still cannot be taken as a comprehensive representation of the actual situation in the state of RJ, as the objective was not to map existing services but to describe the PC services in the state.

This is the first study that characterizes the PC structure in the state of RJ, showcasing the similarities with Brazil and Latin America. This approach provides a comprehensive overview of the strengths and weaknesses of PC services in the state, offering valuable insight to guide interventions aimed at enhancing the efficacy of PC provision in the region.

This study is subject to methodological limitations. Due to the cross-sectional design, some PC services in the state of RJ may have yet to be evaluated and characterized, given the small sample size and the convenience sampling method used.

In addition, several factors can influence the quality of care provided by PC services, such as adequate funding to acquire sufficient resources and personnel, availability of qualified health-care professionals, accessibility for patients in need, cultural barriers, and legal and ethical considerations, including end-of-life care regulations in the region. Therefore, more studies are required to determine whether these findings can be generalized as a comprehensive overview of PC services in the country and across Latin America.

## CONCLUSION

Most of the services evaluated were located in the capital of the state and were mainly composed of hospital-level physicians. The research revealed the fact that the majority of the professionals lacked formal training in PC. Although the pattern of PC in the state of RJ seems to be similar to that of Brazil and Latin America, further scientific studies are necessary to gain a comprehensive understanding of PC services in the country.

## Supplementary Material


