# Supplementary material for: Palliative Care in the state of Rio de Janeiro (Brazil): characteristics of the services
Source: J Med Life. 2023 Aug;16(8):1183–7. doi: 10.25122/jml-2023-0083 (PMC10652673; doi:10.25122/jml-2023-0083)
Supplement: Supplementary file 1 [file JMedLife-16-1183-s001.pdf]

Supplementary Material 1

(fields in * are mandatory)

A1. IDENTIFICATION 1: (WHO FILLS IN)

Full name* ....

Telephone ....

Email* ....

A2. IDENTIFICATION 2: (FROM THE PC SERVICE)

*Name (fantasy and corporate name):

....

*Coordinator — name, contact, and profession:

....

*Full address of headquarters/institution (city and state):

....

*Activities start date (month and year):

....

B. SERVICE CHARACTERISTICS:

*Number of service components and weekly dedication in hours The number of professionals and weekly workload IN SERVICE (Ex: two doctors 20h/week, five nurses/30h weekly, etc.)

- Doctors
How many .... Hours .... are Exclusive to the service? Yes ☐ No ☐

- Nurses:
How many .... Hours .... are Exclusive to the service? Yes ☐ No ☐

- Nursing assistants and technicians:
How many .... Hours .... are Exclusive to the service? Yes ☐ No ☐

- Psychologists:
How many .... Hours .... are Exclusive to the service? Yes ☐ No ☐

- Social Workers:
How many .... Hours .... are Exclusive to the service? Yes ☐ No ☐

Licensed under CC BY 4.0

(fields in * are mandatory)

- Physiotherapists:
How many .... Hours .... are Exclusive to the service? Yes ☐ No ☐

- Occupational therapists:
How many .... Hours .... are Exclusive to the service? Yes ☐ No ☐

- Nutritionists/Dietitians:
How many .... Hours .... are Exclusive to the service? Yes ☐ No ☐

- Phonoaudiologists:
How many .... Hours .... are Exclusive to the service? Yes ☐ No ☐

- Chaplain:
How many .... Hours .... are Exclusive to the service? Yes ☐ No ☐

*Type of Service:
☐ Public ☐ State ☐ Municipal ☐ Federal
☐ YOU
☐ Private
☐ Health insurance/operator
Exclusive to one operator? ☐ NO ☐ YES. Which? ....

*The service:
Does it work in the hospital? ☐ YES ☐ NO
Exclusively? ☐ YES ☐ NO
Do you have your inpatient beds? ☐ YES ☐ NO
Does it work in a tertiary/high-complexity hospital? ☐ YES ☐ NO

*Service mode (check more than one if applicable):
☐ Consultation group (Opinions)
The number of calls/month: ....

☐ Outpatient
The number of calls/month: ....

☐ Inpatient unit within a hospital
The number of calls/month: ....

Average stay (days): ....

☐ Out-of-hospital inpatient unit
The number of beds: ....

Average stay (days): ....

☐ PC-specific home care
The number of calls/month: ....

Average stay (days): ....

☐ Other
Specify mode: ....

Licensed under CC BY 4.0

(fields in * are mandatory)

* Age group served:
Age range (the percentage must complete 100%):
☐ Child/Adolescent
☐ Adult (18 to 59 years old)
☐ Elderly (>60 years)

Condition (the percentage must complete 100%):
☐ Oncology
☐ Non-oncological

*Do you have difficulty accessing opioids in the institution?
☐ YES ☐ NO

*Opioids available (check more than one, if applicable)
☐ Codeine ☐ Oral Tramadol ☐ Parenteral Tramadol ☐ Oral morphine
☐ Parenteral morphine ☐ Oxycodone ☐ Oral methadone ☐ Parenteral methadone
☐ Transdermal Fentanyl ☐ Parenteral Fentanyl

C. TEACHING ACTIVITIES

*Is the service involved with PC education at graduation?
☐ YES ☐ NO

If yes:
☐ Sporadic / by invitation (less than 4 hours per week)
☐ Continued

If continued:
☐ in PC class
☐ in another class

University/Faculty responsible for the pedagogical program:

Is there a Palliative Care Study Group? ☐ YES ☐ NO

*Type of education (check more than one, if applicable)
☐ Graduation in Medicine
☐ Graduation in Nursing
☐ Degree in psychology
☐ Graduation in Social Work
☐ Graduation in another area

*Is the service involved in PC education for residents?
☐ NO
☐ YES
If yes:
☐ Sporadic/by invitation (less than 20 hours per week)
☐ continued

Institution responsible for the pedagogical program:

*Type of education (check more than one, if applicable)

☐ PC medical residency
- Year of commencement of activities: ....

- Number of trained residents: ....

Licensed under CC BY 4.0

(fields in * are mandatory)

☐ Medical residency in a specialty other than PC (at least 80h/month)

- Year of commencement of activities: ....

- Number of trained residents*: ....

- Specialties*: ....

☐ Internal Medicine
☐ Family Medicine
☐ Pediatrics
☐ Geriatrics
☐ Oncology
☐ Anesthesiology
☐ Head and neck surgery
☐ Intensive Care
☐ Infectious diseases
☐ Neurology
☐ Others

☐ Multiprofessional PC residency

- Year of commencement of activities: ....

- Number of trained residents*: ....

- Professions:
☐ Nursing
☐ Psychology
☐ Social service
☐ Physiotherapy
☐ Occupational therapy
☐ Phonoaudiology
☐ Nutrition
☐ Others

☐ Specialized supplementation in PC (at least 20 hours per week in one year)

- Year of commencement of activities: ....

- Number of trained residents*: ....

D. TRAINING OF PROFESSIONALS

*Specific training in PC for professionals (write the number of professionals in each modality below):
☐ Improvement course (at least 180 classroom hours)
☐ Specialization course (at least 360 classroom hours)
☐ Medical or Multiprofessional residency in PC (at least one year, 60 hours per week)
☐ Specialized supplementation in PC (at least one year 30 hours per week)
☐ Training abroad (observership) - at least one year
☐ In-service (hands-on) training abroad - at least one year
☐ In-service (hands-on) training abroad - between 3 months and one year
☐ In-service (hands-on) training abroad - less than three months.
☐ No specific training

Licensed under CC BY 4.0

(fields in * are mandatory)

E. OTHER DATA:

*Are there volunteers?

☐ YES ☐ NO If YES, how many? ....

* Are there any regular and scheduled activities specifically aimed at caring for the team?

☐ YES ☐ NO

* Are there any regular and scheduled activities specifically aimed at caring for the family or informal caregiver?

☐ YES ☐ NO

*Does the service rely on regular donations of financial resources?

☐ YES ☐ NO

Licensed under CC BY 4.0
